# Understanding the treatment of allergic rhinitis

**DOI:** 10.1016/S1808-8694(15)30592-9

**Published:** 2015-10-18

**Authors:** João Ferreira de Mello Júnior

**Affiliations:** Associate Professor – University of São Paulo Medical School

**Keywords:** allergic, physiopathology, perennial, rhinitis

At this time of the year, in some Brazilian regions (Southeast and Mid-West), the weather becomes very dry, and in some cities, the level of air pollution is substantially bad. These factors, amongst others, contribute to a worsening in the symptoms of those patients with inflammatory rhinitis, and they may become predisposed to developing infectious rhinitis.

According to ARIA (Allergic Rhinitis And Its Impact on Asthma), rhinitis is an inflammatory process that affects the nasal mucosa, causing nasal obstruction, anterior and/or posterior rhinorrhea, sneezing and nasal pruritus, occurring for more than two consecutive days and lasting for more than one hour in most days.

In allergic rhinitis, the inflammatory process starts with the Type I reaction of Gell and Coobs's classification. In genetically predisposed individuals, they will produce immunoglobulin E (IgE) for some antigens. The interaction between these antibodies (linked to mast cells) and the antigens results in the release of numerous mediators (histamine, leukotrienes, etc.) which act on receptors to trigger the symptoms. Moreover, some of them are capable of attracting inflammatory cells (eosinophils) for the region (nasal mucosa), which will be activated and will release their mediators (eosinophillic cationic protein, eosinophillic peroxidase, etc.), responsible for local inflammation.

Physiologically speaking, nasal breathing has a cycle, while one of the nasal cavities is more resistant to air flow (obstructed), the other remains patent. The stimuli responsible for vasodilatation also cause increase in glandular secretion and stimulate cilia beating. After a few hours (2 to 6) the one that was obstructed starts to receive vasoconstriction stimuli, and vice-versa. This physiological nasal cycle is controlled by the autonomic nervous system (ANS), which is altered by rhinitis. Such patients have a local sympathetic hyporeactivity and parasympathetic hyperactivity, consequently presenting symptoms caused by unspecific stimuli (changes in temperature or in air moisture, etc).

In patients with allergic rhinitis we also find neurogenic inflammation. Some neuropeptides secreted by the ANS (NPY) cause the release of interleukins, which are directly associated with allergies, such as IL-3, IL-4 and IL-5. While IL-4 is important in inducing the expression of adhesion molecules associated with eosinophil attraction, IL-3 and 5 are responsible for its activation and apoptosis reduction.

**Figure 1:**
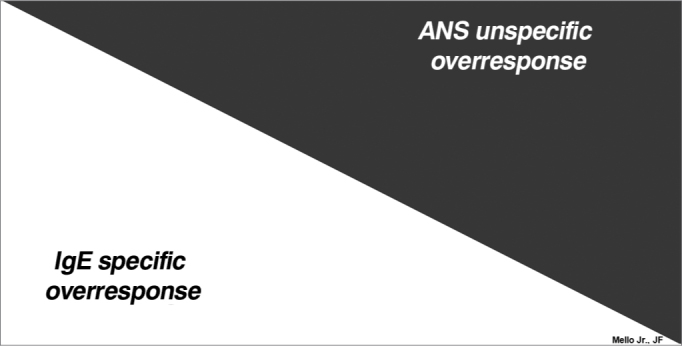
Legend: relevance of allergy and autonomic nervous system mechanisms in the cause of symptoms of patients with allergic rhinitis.

Understanding such concepts can help treat patients with allergic rhinitis. In other words, since they simultaneously present local hyperactivity towards specific and unspecific agents (Figure 1), the physician's role is to identify the relevance of each of these mechanisms that cause symptoms. This must be considered when one chooses the drug treatment, because if IgE-mediated symptoms prevail, the response to anti-histamines and chromones, for example, will be better than in those cases in which ANS hyperactivity is more important.

